# Splicing factor 3b subunit 1 (*Sf3b1*) haploinsufficient mice display features of low risk Myelodysplastic syndromes with ring sideroblasts

**DOI:** 10.1186/s13045-014-0089-x

**Published:** 2014-12-07

**Authors:** Valeria Visconte, Ali Tabarroki, Li Zhang, Yvonne Parker, Edy Hasrouni, Reda Mahfouz, Kyoichi Isono, Haruhiko Koseki, Mikkael A Sekeres, Yogen Saunthararajah, John Barnard, Daniel Lindner, Heesun J Rogers, Ramon V Tiu

**Affiliations:** Department of Translational Hematology and Oncology Research, Taussig Cancer Institute, Cleveland Clinic, 9500 Euclid Avenue R40, Cleveland, OH USA 44195; Department of Medicine, University of California, School of Medicine, San Francisco, CA USA; Center for Integrative Medical Sciences (IMS), RIKEN, Yokohama Institute, Yokohama, Japan; Leukemia Program, Department of Hematology and Oncology, Taussig Cancer Institute, Cleveland Clinic, Cleveland, OH USA; Department of Quantitative Health Sciences, Cleveland Clinic, Cleveland, OH USA; Department of Laboratory Medicine, Cleveland Clinic, Cleveland, OH USA

**Keywords:** *SF3B1* mice, Myelodysplasia, RNA-sequencing

## Abstract

**Background:**

The presence of somatic mutations in splicing factor 3b subunit 1 (*SF3B1*) in patients with Myelodysplastic syndromes with ring sideroblasts (MDS-RS) highlights the importance of the RNA-splicing machinery in MDS. We previously reported the presence of bone marrow (BM) RS in *Sf3b1* heterozygous (*Sf3b1*^+/−^) mice which are rarely found in mouse models of MDS. *Sf3b1*^+/−^ mice were originally engineered to study the interaction between polycomb genes and other proteins.

**Methods:**

We used routine blood tests and histopathologic analysis of BM, spleen, and liver to evaluate the hematologic and morphologic characteristics of *Sf3b1*^+/−^ mice in the context of MDS by comparing the long term follow-up (15 months) of *Sf3b1*^+/−^ and *Sf3b1*^+/+^ mice. We then performed a comprehensive RNA-sequencing analysis to evaluate the transcriptome of BM cells from *Sf3b1*^+/−^ and *Sf3b1*^+/+^ mice.

**Results:**

*Sf3b1*^+/−^ exhibited macrocytic anemia (MCV: 49.5 ± 1.6 vs 47.2 ± 1.4; Hgb: 5.5 ± 1.7 vs 7.2 ± 1.0) and thrombocytosis (PLTs: 911.4 ± 212.1 vs 878.4 ± 240.9) compared to *Sf3b1*^+/+^ mice. BM analysis showed dyserythropoiesis and occasional RS in *Sf3b1*^+/−^ mice. The splenic architecture showed increased megakaryocytes with hyperchromatic nuclei, and evidence of extramedullary hematopoiesis. RNA-sequencing showed higher expression of a gene set containing *Jak2* in *Sf3b1*^+/−^ compared to *Sf3b1*^+/+^.

**Conclusions:**

Our study indicates that *Sf3b1*^+/−^ mice manifest features of low risk MDS-RS and may be relevant for preclinical therapeutic studies.

**Electronic supplementary material:**

The online version of this article (doi:10.1186/s13045-014-0089-x) contains supplementary material, which is available to authorized users.

## Background

Myelodysplastic syndrome (MDS) is a heterogeneous group of hematopoietic stem cell disorders characterized by peripheral blood (PB) cytopenias, dysplastic bone marrow (BM), and increased risk of transformation to acute myeloid leukemia (AML). Within MDS, refractory anemia with ring sideroblasts (RARS) is a low-grade disease characterized by anemia, erythroid dysplasia, and the presence of 15% or more RS [1]. Some patients with RARS also present with marked thrombocytosis (RARS-T), a form of myelodysplastic/ myeloproliferative neoplasm (MDS/ MPN) associated with mutations in *JAK2*, *TET2*, and *MPL* genes [2-5]. The presence of RS is a key pathologic criterion for the diagnosis of both RARS and RARS-T. RS are erythroblasts with an abnormal localization of mitochondrial iron which appears in the shape of a blue ring by light microscopy. Studies investigating the mechanisms of RS formation in MDS implicated the mitochondrial genes *ALAS2* and *ABCB7* based on the gene expression differences detected in CD34-positive cells of RARS and RARS-T patients compared to healthy individuals [6,7]. The discovery of recurrent somatic mutations in splicing factor 3b, subunit 1 (*SF3B1*), a component of the RNA splicing machinery in approximately 60% of RARS and 82% of RARS-T patients opened a new area of study in MDS [8-12].

*SF3B1* is a core component of the U2 small nuclear ribonucleoprotein (U2 snRNP). The function of *SF3B1* is to recognize the 3’ splice site at the intron-exon boundaries of pre-nascent RNAs. SF3B1 protein interacts with the 3′-splice-site recognition of U2AF65 and other splicing factors such as SF3B14 to facilitate the successive steps of RNA splicing [13,14]. Although *SF3B1* has been associated with MDS-RS, the biological role and the functional consequences of the genetic alterations in this gene on the pathogenesis of MDS-RS have not been fully elucidated. We previously reported that a mouse model characterized by *Sf3b1* haploinsufficiency (*Sf3b1*^+/−^) have RS in the BM [15]. Isono *et al.* generated the *SF3B1*^*+/−*^ mice by replacing 4 exons of *Sf3b1* (chromosome 1qC1.2) with a neo-cassette to investigate the interaction of Sf3b1 protein with the polycomb group of proteins*.* In 2005, they reported that *Sf3b1*^−/−^ mice were embryonic lethal whereas *Sf3b1*^+/−^ mice survived and exhibited several skeletal abnormalities [16]. However, the long-term dynamics of the hematologic phenotype of this mouse model was not analyzed. The diagnosis of human MDS is strictly based on blood counts, BM morphology, and cytogenetic criteria. Similarly, the criteria established by the *Mouse Models of Human Cancers Consortium* are also only weighted on PB counts and morphologic features*.* It is for this reason that we focused our investigation on the long term PB and BM morphologic characteristics of *Sf3b1*^+/−^ mice to help establish if this mouse model displays features of MDS and can therefore serve as a robust mouse model to study human RARS and RARS-T and a platform to test new therapies.

## Results

### Genomic analysis of *Sf3b1* mice

Embryos of the *Sf3b1* mice were purchased from RIKEN. Mating of *Sf3b1* mice was conducted in-house at the Cleveland Clinic. All procedures were approved by the Institutional Animal Care and Use Committee (IACUC) of the Cleveland Clinic.

None of the *Sf3b1*^+/−^ or *Sf3b1*^+/+^ mice died immediately after birth and no obvious skeletal abnormalities were noted. There were no reported early deaths in either cohort. A total of 78 mice were analyzed (*Sf3b1*^+/−^/*Sf3b1*^+/+^ = 33/45). There were no homozygous *Sf3b1*^−/−^ mice. Tissues from tail and toes were taken in the first 10 days of life and used as a source of genomic DNA. PCR analysis showed that *Sf3b1*^+/−^ mice carried 2 PCR products: a wild type (WT) band at 1.5 kb and knock-out (KO) band at 0.9 kb as shown for mice # 1, 2, 4, and 7 in Additional file 1: Figure S1.

### Hematologic findings of *Sf3b1*^+/−^ mice

Mouse models of MDS demonstrate specific features resembling human MDS disease albeit at variable time points [17]. This fact underlines the importance of long term follow-up of mouse models to accurately capture disease-related events. We examined the standard hematologic parameters of *Sf3b1*^+/−^ (n = 5) and *Sf3b1*^+/+^ (n = 5) starting from 6 months of age every month. After 6 months of age, fertility of breeding pairs dropped dramatically. No progeny was produced by mice of this age or older. The mechanism for this decline is unclear at present and it is under active investigation.

**Figure 1:**
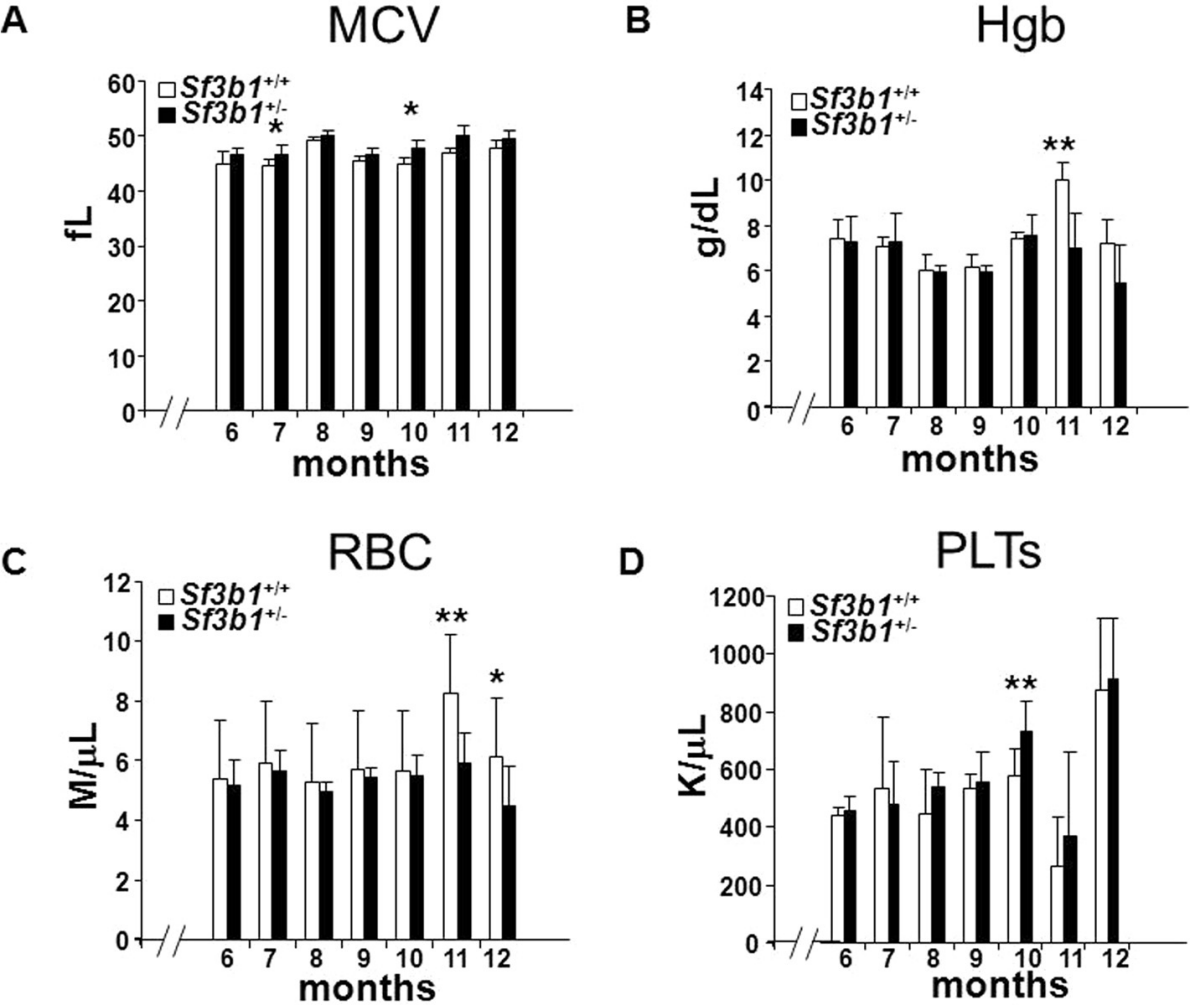
**Hematologic parameters in** ***Sf3b1*** ^**+/−**^ **compared to** ***Sf3b1*** ^**+/+**^ **mice.** Complete blood cell count (CBC) was measured during long-term follow-up of *Sf3B1* ^+/+^ (n = 5; Female/Male = 3/2) and *Sf3b1* ^+/+^ (n = 5; Female/Male = 3/2) mice. Blood was taken every month and measured using a Hemavet 500 instrument until 12 months of age. *Sf3b1* ^+/+^ are indicated in white and *Sf3b1* ^+/−^ in black bars, respectively. Data are presented as mean ± standard deviation for mean corpuscular volume (MCV) **(A)**, hemoglobin (Hgb) **(B)**, red blood cells (RBC) **(C)** and platelets (PTL) **(D)**. *Indicates a significant difference (*P* ≤ 0.05). **Indicates a significant difference (*P* ≤ 0.01). -//- indicates the interval between 0 and 6 months of age.

In terms of MCV, the *Sf3b1*^+/−^ mice have a higher MCV compared to *Sf3b1*^+/+^ at 6 (46.72 fL ± 1.32 vs 44.98 fL ± 2.32) to 12 months (49.50 fL ± 1.58 vs 47.68 fL ± 1.40) of age. Levels of statistical significance were reached at 7 (*P* = 0.047) and 10 (*P* = 0.031) months of age (Figure 1A).

In terms of Hgb levels, *Sf3b1*^+/−^ mice tend to have lower values compared to *Sf3b1*^+/+^ at 6, 8, 9, 11, and 12 months of age. Statistically significant difference was noted at month 11 (6.97 g/dL ± 1.60 vs 10.04 g/dL ± 0.73; *P* = 0.008) of age (Figure 1B). As expected, the trend of the RBC values paralleled the trend of the Hgb levels with statistical significance being reached at 11 (5.96 M/uL ± 1.02 vs 8.28 M/uL ± 0.48; *P* = 0.008) and 12 (4.52 M/uL ± 1.30 vs 6.09 M/uL ± 0.82; *P* = 0.027) months of age (Figure 1C). PLT counts increased at month 6 until month 12 of age with a significant difference at month 10 (731 K/uL ± 105.36 vs 579 K/uL ± 92.66; *P* = 0.008) (Figure 1D). We also observed that after 12 months of age, some of the mice (n = 3) started to show a decline in overall activity characterized by reduced movements and difficulty walking which culminated in death a few weeks later. Two of the deaths were in the *Sf3B1*^+/+^ group while 1 occurred in the *Sf3b1*^+/−^ cohort.

Since somatic heterozygous mutations in *SF3B1* were also identified in a specific cohort of chronic lymphocytic leukemia (CLL) patients [18] we also measured and analyzed the leukocyte counts of the mice. The leukocyte compartment was primarily enriched with lymphocytes. However, the values were variable over time in both mice groups (data not shown). Moreover, mast cells were also evaluated in BM cells derived from *Sf3b1*^+/−^ (n = 2) and *Sf3b1*^+/+^ (n = 2) by performing immunohistochemistry for CD117 (c-Kit) (Additional file 2: Figure S2). Mast cells noted as CD117 positive cells were rare and scattered and no difference was detected between both groups.

### Histological examination of bone marrow, spleen, and liver

**Figure 2:**
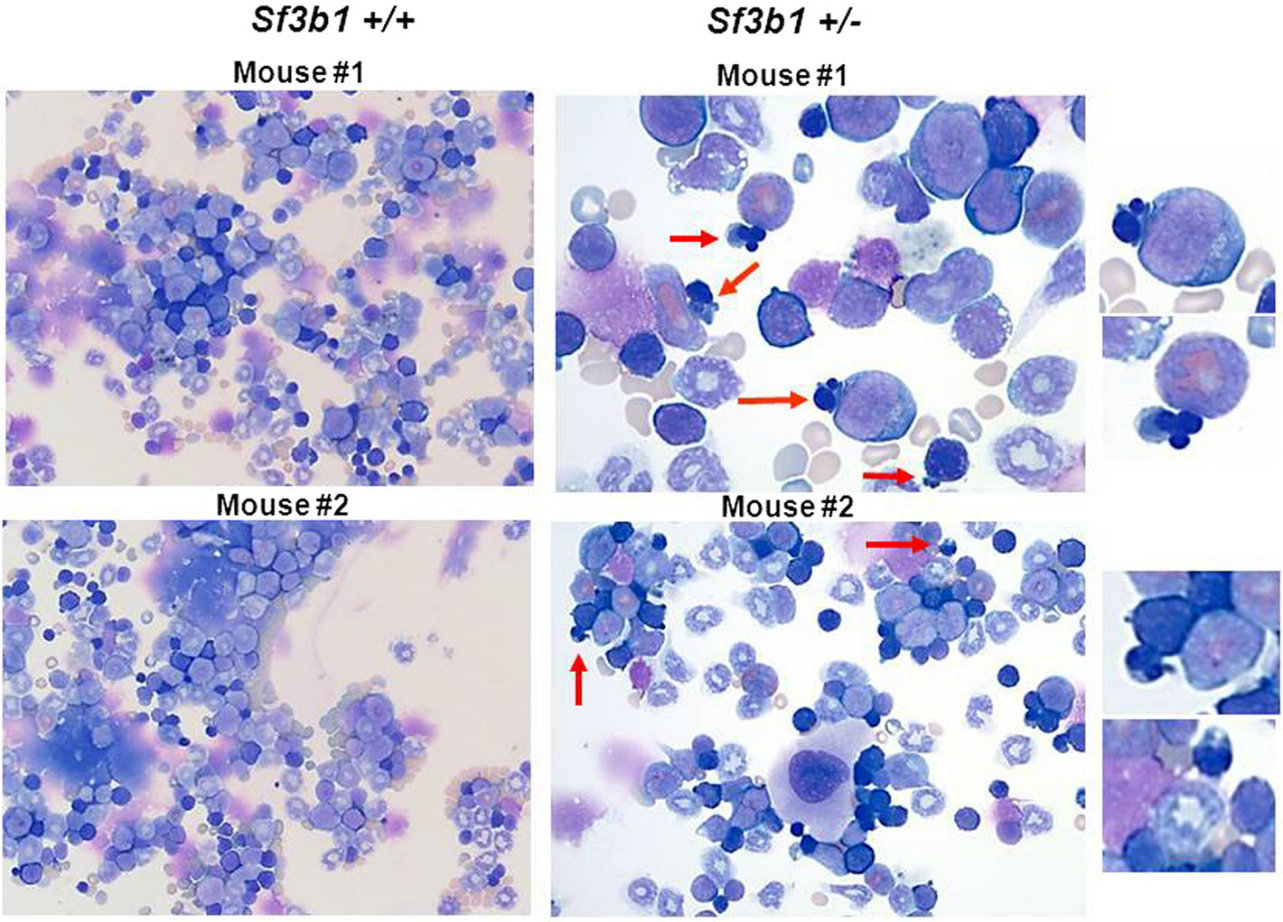
**Bone marrow morphology in** ***Sf3b1*** ^**+/−**^ **compared to** ***Sf3b1*** ^**+/+**^ **mice.** Bone marrow (BM) cells were extracted by flushing femurs of *Sf3b1* ^+/−^ (n = 5) and *Sf3b1* ^+/+^ (n = 5) in media supplemented with 10% fetal bovine serum. Cells (2–3 x 10^5^) were washed and spotted on cytospin slides prior immersion in buffered Wright-Giemsa staining solution. Budding and irregular nuclei are indicated in red arrows and are also magnified in the right quadrants. This feature was also observed in slides of BM cells from *Sf3b1* ^+/−^ mice subjected to iron staining (Additional file 4: Figure S4). The image is presented for 1 Sf3b1+/- mouse.

**Figure 3:**
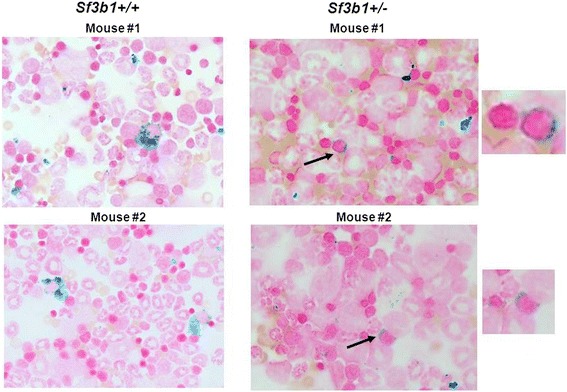
**Detection of ring sideroblasts by Prussian blue staining in** ***Sf3b1*** ^**+/−**^ **compared to** ***Sf3b1*** ^**+/+**^ **mice.** Bone marrow cells were extracted from femurs of *Sf3b1* ^+/−^ (n = 5) and *Sf3b1* ^+/+^ (n = 5) and cells (2-3x10^5^) spotted on cytospin slides prior staining with Prussian blue. Ring sideroblasts (RS) were detected in *Sf3b1* ^+/−^ compared to *Sf3b1* ^+/+^ mice. Images were taken from 2 mice per group. RS were also detected in additional mice as shown in Additional file 5: Figure S5.

We first examined and compared the BM cellularity between the *Sf3b1*^+/−^ and *Sf3b1*^+/+^ mice. H&E stain showed trilineage hematopoiesis with an adequate number of megakaryocytes in both groups of mice (Additional file 3: Figure S3, panel A). No difference in the number of BM cells was also observed at the end of the study between *Sf3b1*^+/−^ and *Sf3b1*^+/+^ mice (57.9 ± 9.5 vs. 60.2 ± 8.4; *P* = 0.74) (Additional file 3: Figure S3, panel B). We next examined the morphology of BM cells derived from *Sf3b1*^+/−^ and *Sf3b1*^+/+^ mice. BM (2–3 × 10^5^) cells were spotted on cytospin slides and stained with Wright-Giemsa. BM cells from *Sf3b1*^+/−^showed dyserythropoietic features including nuclear budding or nuclear irregularity (Figure 2, red arrows) similar to what is observed in human MDS. Similar features were also noted in slides stained with Prussian blue (Additional file 4: Figure S4, black arrows). We originally reported the presence of rare RS in BM slides from *Sf3b1*^+/−^ mice [15]. We confirmed this observation by performing Prussian blue staining on fresh BM cytospin slides and finding occasional RS (Figure 3, black arrows) in the BM of *Sf3b1*^+/−^ mice while BM cells from *Sf3b1*^+/+^ only showed iron accumulation in histiocytes. RS were noted in several BM slides as shown in Additional file 5: Figure S5, black arrows.

**Figure 4:**
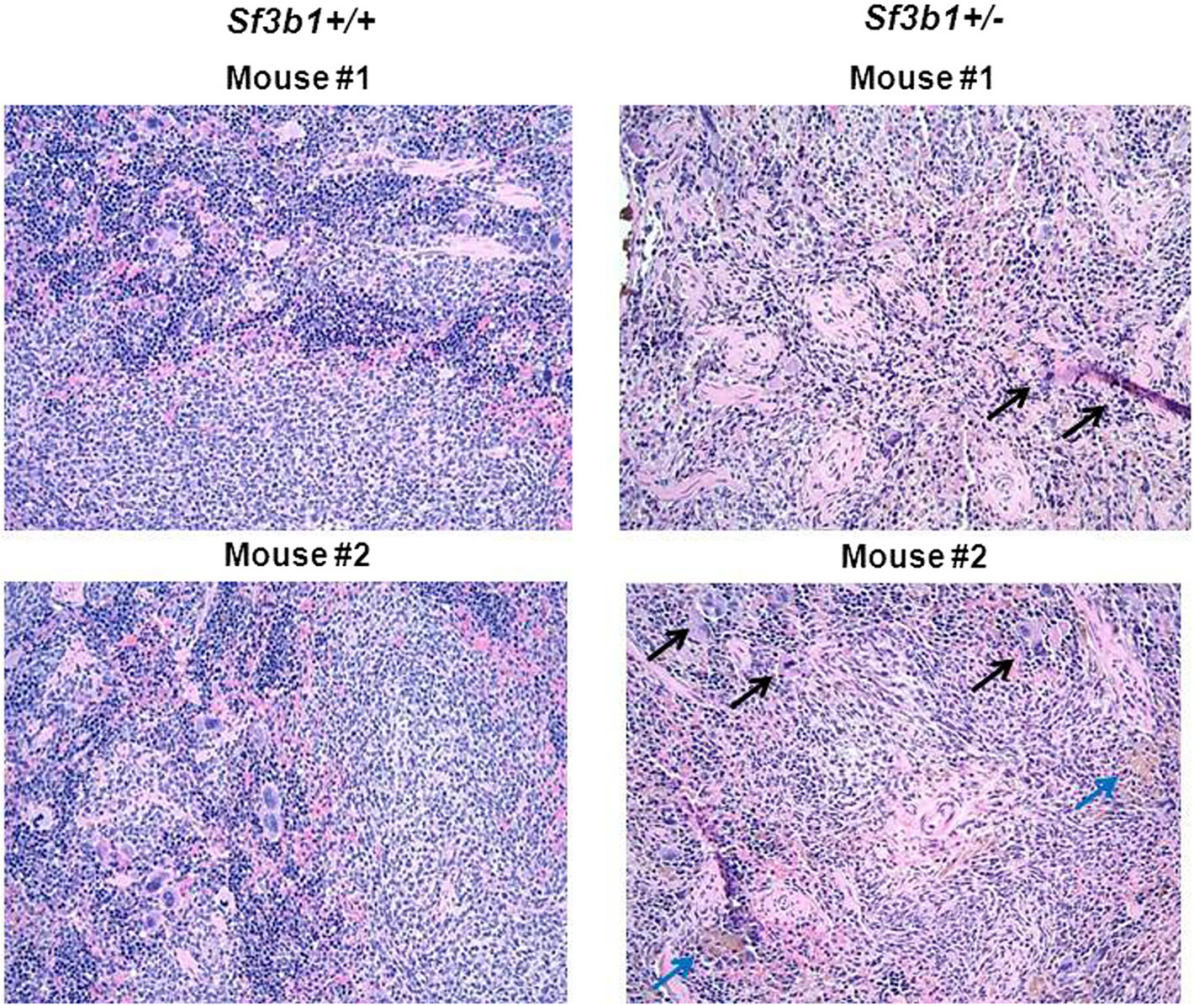
**Histology of splenic tissues of** ***Sf3b1*** ^**+/−**^ **compared to** ***Sf3b1*** ^**+/+**^ **mice.** Spleens from *Sf3B1* ^+/−^ and *Sf3b1* ^+/+^ mice were fixed in 4% formaldehyde/PBS and embedded in paraffin. Sections were stained with Haematoxylin–Eosin and showed extramedullary hematopoiesis with all 3 hematopoietic elements, increased megakaryocytes with hyperchromatic nuclei (black arrows), increased hemosiderin deposits (blue arrows) and evidence of fibrosis.

The spleen and liver from both groups of mice were also dissected, measured, and histopathologically examined at the end of the study. Spleen and liver weights were compared between *Sf3b1*^+/−^ (n = 4) and *Sf3b1*^+/+^ (n = 3) (0.10 ± 0.02 vs. 0.08 ± 0.01; *P* = 0.08; 1.13 ± 0.15 vs. 1.36 ± 0.21; *P* = 0.14). Microscopic examination of the spleen tissues showed significant expansion in the red pulp of *Sf3b1*^+/−^ mice with finding of extramedullary hematopoiesis (EMH) with hematopoietic elements, increased megakaryocytes with hyperchromatic nuclei, increased hemosiderin deposits and signs of fibrosis (Figure 4) but no hepatomegaly or microscopic abnormalities in the liver were noted (Additional file 6: Figure S6).

### RNA-sequencing analysis showed overexpression of Jak2 and other hematopoietic-related gene sets

We performed RNA-sequencing to characterize and compare the transcriptome profile of BM cells derived from 2 female *Sf3b1*^+/−^ and 2 female *Sf3b1*^+/+^ mice. Total 100-bp reads (mapped to mm10 genome reads) in millions for the four mice were 35.54 (22.29), 31.18 (18.50), 35.07 (21.09) and 48.48 (26.28), of which 17.69, 15.54, 16.70 and 22.52 million reads, respectively, mapped to 20,207 mouse genes. After filtering by gene intensity, 17.67, 12.46, 16.67 and 22.49 million reads, respectively, mapped to 10,330 genes. Global gene level differential expression analysis of these 10,330 genes did not find any significant differential expression in *Sf3b1*^+/−^ compared to *Sf3b1*^+/+^ mice (Additional file 7: Table S1). The target gene *Sf3b1* showed evidence of down-regulation [fold change (FC) = 0.75; *P* = 0.075, rank = 309] in *Sf3b1*^+/−^ vs *Sf3b1*^+/+^ mice. Since this *Sf3b1*^+/−^ mouse model was originally developed to study the interaction of Sf3b1 protein and proteins of the polycomb (PcG) complex, we also evaluated the status of known PcG genes, finding a trend towards lower mRNA levels of Ezh2 (FC = 0.02; *P =* 0.185*,* rank = 1387). We also found higher mRNA levels of Bmi1 (FC = 1.69; *P* = 0.138, rank = 471), a component of the PcG repressive complex, which is involved in axial skeletal development. This is likely a consequence of repression of the *Hox* genes. Bmi1 has been associated with progressive loss of proliferative capacity of hematopoietic stem cells and anemia. In addition, gene expression analysis of genes important in MDS pathogenesis showed weak evidence for lower mRNA expression levels of Npm1 (FC = 0.01; *P* = 0.184, rank = 1363) and no evidence of changes for Asxl1 and Runx1 (FC = 1.25; *P* = 0.296 and FC = 1.21; *P* = 0.471) in *Sf3b1*^+/−^ vs *Sf3b1*^+/+^.

Because in human MDS, *SF3B1* clones are found in early hematopoietic stem cells, [19] we interrogated gene sets and genes related to hematopoietic stem cell function and signaling. In total 39 gene sets were selected from the MSigDB c2 collection (gene set results in Additional file 8: Table S2; gene results for members of the gene sets in Additional file 9: Table S3) and showed that hematopoietic receptors mainly expressed in myeloid cells like Trem1 and transcriptional factors involved in hematopoietic development like Ptsg2 (Cox2) were over-expressed in *Sf3b1*^+/−^ (FC = 2.80, *P =* 0.011 and 2.43, *P =* 0.028). Thrombospondin-1 (Thbs1), a glycoprotein involved in the *in-vitro* proliferation of megakaryocytes was also one of the highest ranked genes and was found to be over-expressed (FC = 2.67, *P =* 0.008). Haploinsufficiency of Nr4a1 and Nr4a3, two nuclear receptors expressed in hematopoietic stem and myeloid cells, has been shown to cause MDS/MPN and leukemic evolution in mice [20]. In patients with MDS carrying *SF3B1* mutations, the risk of AML transformation is less compared to those with WT *SF3B1*. In this study, Nr4a1 was found to be over-expressed in *Sf3b1*^+/−^ mice compared to *Sf3b1*^+/+^ mice (FC = 2.29, *P* = 0.038) but Nr4a3 was not detected. We also observed some evidence of down-regulation in Sh2b3 (Lnk) and Calr in *Sf3b1*^+/−^ (FC = 0.22, *P* = 0.261 and FC = 0.33, *P* = 0.193) compared to *Sf3b1*^+/+^ mice. Mutations in both genes have been found in human MPNs. In addition, a Stat5 target gene set showed some evidence of increased expression in *Sf3b1*^+/−^ mice (gene set *P* = 0.064).

From our global gene set analysis of collections c1 through c7, we found 1 significant gene set in the human chromosomal location collection (c1), chr9p24 (gene set *P* = 0.00032). This gene set contained Jak2 and showed higher expression in *Sf3b1*^+/−^ compared to *Sf3b1*^+/+^ mice. We also evaluated genes associated with mitochondrial function (*Abcb7*, *Alas2*, and *Sod2*), which may possibly explain the anemia phenotype in *Sf3b1*^+/−^ mice. Only *Abcb7* showed a weak evidence of increased expression in *Sf3b1*^+/−^ mice (FC = 1.58, *P* = 0.096).

## Discussion

MDS is a heterogeneous disease with a variety of clinical, morphologic, and biological features. Mouse models may provide helpful insight into the mechanisms whereby specific genetic alterations can contribute to disease pathogenesis and can serve as platforms to study therapies that may be useful to treat these diseases. In MDS, the Human Cancer Consortium Mouse Model Group established the set of criteria that defines a MDS mouse model. There are 3 main criteria including the presence of at least one PB cytopenia (anemia, neutropenia or thrombocytopenia), the presence of a maturation arrest in a non-lymphoid hematopoietic component demonstrated in the form of dysplasia, and the absence of criteria of a non-lymphoid leukemia [17,21]. Here we report the hematologic and some of the biologic characteristics of a mouse model with *Sf3b1* haploinsufficiency. The *Sf3b1*^+/−^ mice demonstrated macrocytic anemia, thrombocytosis, dyserythropoiesis, RS and EMH in the spleen. These are findings clinically and pathologically observed in human RARS and RARS-T. We also observed that PLT levels were increased and the spleen was enlarged as demonstrated by EMH in *Sf3b1*^+/−^ which are important clinical features of RARS-T patients. In humans, somatic mutations in *JAK2* have been associated with disorders characterized by increased number of PLTs like RARS-T and related MPNs [5]. Patients with *JAK2* mutations are also frequently found to have an enlarged spleen and an evidence of EMH. Definitive evidence of RS in erythroid precursors were once again consistently identified although in small numbers in the *Sf3b1*^+/−^ and not in the *Sf3b1*^+/+^ mice supporting our initial report that demonstrated rare RS in this mouse model. Somatic mutations in *SF3B1* have also been found in 7-15% of CLL patients and associated with aggressive phases of the disease, relapsed and chemorefractory CLL [18]. The link between *SF3B1* mutations and CLL pathogenesis remain unclear. In MDS, mutations have been associated with a better survival outcome and a lower rate of AML transformation. Interestingly, we noticed an enrichment of the lymphocyte compartment in our mouse model although the increase was variable over time. Studies of *Sf3b1* haploinsufficiency identified a reduction of hematopoietic stem cell pool confined in the myeloid compartment. Our data differ from a recent paper where *Sf3b1*^+/−^ haploinsufficiency appears to only lead to an impairment in the stem cell function but does not lead to MDS features in the same mouse model [22]. Matsunawa *et al.* investigated the functional role of *Sf3b1* in normal hematopoiesis in this mouse model describing that besides a decrease in the number of hematopoietic cells and a reduced capability of hematopoietic reconstitution, no features of MDS were observed. Based on their results there was no change in the number of WBC and PLTs and in the content of Hgb up to 44 weeks (Additional file 1: Figure S1, panel A) [22]. Morphologically, Matsunawa *et al.* did not detect any RS and any change in spleen size by weight estimation. Although, the same mouse model was used, there are key differences in the methodology that significantly affected the outcomes of both studies. Our current study aimed to study specifically the morphologic features of this mouse model using conventional routine techniques used in the assessment of clinico-pathologic features of human MDS and MPN and during long term follow-up. This is an important difference since some mouse models exemplified by Sall4 (14.5 months), Evi1/Evi1t (12 months), NPM-1 (6–18 months) and Arid4a (12–22 months) did not show their respective phenotypes until the mouse models were much older and had longer follow-up [17]. This is in keeping with human MDS, where the vast majority of patients are diagnosed at an elderly age with a median age of diagnosis of 71 years old [23]. Next Matsunawa *et al.* did not analyze specifically the dysplastic morphologies and no images of cellular morphology of the BM aspirates have been shown. The tabulated hematologic results presented in their study showed a lower percentage of erythroid cells in *Sf3b1*^+/−^ mice compared to *Sf3b1*^+/+^ further supporting our findings (*P* = 0.07). The histomorphologic features of the spleen, a frequently affected organ in human RARS-T were also not studied in the prior study. Our study showed that the spleen of the *Sf3b1*^+/−^ was not just enlarged but displayed architectural changes consistent with EMH akin to patients with human RARS-T. The RNA-sequencing results also support the fact that *Sf3b1*^+/−^ mice have a pattern more close to low rather than to high-risk MDS. In human MDS, *ASXL1* mutations have been found enriched in patients with high-risk rather than in low-risk MDS and are correlated with unfavorable outcomes and AML transformation. In addition patients with *ASXL1* mutations carry concomitant *RUNX1* mutations and lower incidence of *SF3B1* mutations [24]. Studies in mice showed that *Asxl1* haploinsufficiency leads to a reduced hematopoietic stem cell pool, decreased hematopoietic repopulating capacity, and mild features of MDS [25]. On the same line, mice expressing the *RUNX1* frameshift mutation (S291fs) develop signs of MDS including excess of blasts and dysplasia of the erythroid compartment [26]. In our mouse model, we observed minimal changes in the expression levels of both *Asxl1* and *Runx1,* factors traditionally associated with more inferior outcomes in patients with MDS further supporting the natural history of human RARS-T probably due to the fact that *Sf3b1*^+/−^ mice do not manifest a late stage higher risk MDS disease. Indeed we did not observe any increased in blasts percentage and any sign of AML development despite the long term follow-up.

Our clinicopathologic results are further supported by RNA sequencing analysis where we found an over-expression of Jak2 and a down-regulation of Sh2b3 and Calr mRNA levels consistent with what is observed in human RARS-T. In regards to RS we consistently identified RS in the BM of these mice by using two blinded independent hematopathologists and this is unlikely to be simply a matter of chance. Lastly, using the guidelines established by the hematopathology subcommittee of the *Mouse Models of Human Cancers Consortium*, [17,21], it clearly shows that this mouse model fulfills the criteria for an MDS mouse model (Additional file 10: Figure S7).

## Conclusions

In conclusion, our current data show that *Sf3b1* haploinsufficiency in mice causes biological and morphological features resembling low risk MDS patients with RS specifically RARS and RARS-T opening the possibility that this mouse model can be helpful in testing therapeutic approaches in low risk MDS.

## Methods

### Mice

All procedures were approved by the Institutional Animal Care and Use Committee (IACUC) of the Cleveland Clinic. *Sf3b1*^+/−^ mice were originally developed by Isono *et al.* [16] Cryopreserved embryos of *Sf3b1*^+/−^ mice were purchased from Dr. H. Koseki and Dr. K. Isono from the Center for Integrative Medical Sciences (IMS) RIKEN (Japan) in early 2012. Embryos were successfully implanted in foster mothers and rederived mice were genotyped.

### Genotyping

DNA derived by tail and toe clippings was extracted using a Puregene Core kit A (Qiagen, Valencia, CA) following the manufacturer’s instruction. DNA (100 ng) was used for PCR amplification using 3 sets of primers: primer #1 [specific for the *neo* gene (5′ GCGTGCAATCCATCTTG’)], primer #2 [specific for *Sf3b1* (5′ AAGAATTCGTCATTGACACTTTTCA)], and primer #3 [specific for *Sf3b1* (5′ GACTGAGCTCAGATAACATG)]. PCR conditions were: initial denaturation at 98°C for 1 min, 35 cycles (94°C for 1 min, 60°C for 1 min, 72°C for 2 min) and a final extension at 72°C for 7 min. PCR products were resolved on 1.2% agarose gels. Gel micrographs were acquired using a Quantity One 1D-analysis software (Bio-Rad Laboratories, Hercules, CA).

### Long-term evaluation of *Sf3b1*^+/−^ mice

A total of 5 *Sf3b1*^+/−^ (3 females/ 2 males) and 5 *Sf3b1*^+/+^ (3 females/ 2 males) were maintained on a regular diet. Blood was collected by retro-orbital puncture in heparinized tubes every month. Blood was diluted 1:1 with PBS containing 2.7 mM EDTA and standard blood parameters [leucocyte counts, mean corpuscular volume (MCV), red blood cells (RBC), hemoglobin (Hgb), and platelets (PLTs)] were measured using a Hemavet 950 FS analyzer (Drew Scientific Incorporation, Dallas, TX). Mice were sacrificed at the endpoint of the study and tissues were collected as following: femurs from 2 mice per genotype were submitted for hematoxylin and eosin (H&E) stain, BM from all mice was flushed with Iscove’s Modified Dulbecco’s media plus 10% fetal bovine serum using a 25-gauge needle syringe from femurs and evaluated for cell count using a Vi-Cell™ XR cell viability analyzer (Beckman Coulter, Brea, CA). The spleen and liver were also fixed in 4% formaldehyde/PBS and stained with H&E.

### Histomorphological analysis and Prussian blue staining

BM cells from femurs of *Sf3b1*^+/−^ and *Sf3b1*^+/+^ mice were flushed with Iscove’s Modified Dulbecco’s medium supplemented with 10% fetal bovine serum (FBS). Cells (2×10^5^) were washed once with PBS supplemented with 2% FBS and spotted on cytospin slides before Wright-Giemsa and Prussian blue stains were performed using standard histopathology staining procedures. Spleen and liver from *Sf3b1*^+/−^ and *Sf3b1*^+/+^ mice were fixed in 4% formaldehyde/PBS and embedded in paraffin before H&E staining.

### RNA Sequencing (RNA-Seq) analysis

#### Mapping

Total RNA was extracted from whole BM of 6-month-old female *Sf3b1*^+/−^ (n = 2) and *Sf3b1*^+/+^ (n = 2) mice using NucleoSpin RNA II (Clontech Laboratories). PolyA cDNA was prepared from 3 μg of RNA and mouse RNA-sequencing was run on Illumina HiSeq2000 by Otogenetics (Norcross, GA). 100 basepair paired-end RNA-sequencing reads were mapped to the mm10 RefSeq mouse transcriptome and spliceome by DNAnexus (http://dnanexus.com) using a Bayesian method where a read was mapped when its posterior probability of mapping exceeded 0.9. These filtered posterior probabilities were summed to generate fractional read counts per gene and per exon, with probabilities from splice-junction spanning reads counted for each relevant exon. We used rounded gene and exon read counts as inputs for our differential expression analyses.

#### Differential gene expression analysis

We used TMM [27] normalization and the voom-limma approach [28] from the R package limma version 3.17 with R version 3.0.1 in order to perform differential gene expression analysis for *Sf3b1*^+/−^ versus *Sf3b1*^+/+^ samples. Before testing, we dropped all genes with read counts per million reads less than or equal to 1 in at least 2 samples to improve testing power while maintaining type I error rates. We used the lmFit function with empirical Bayes shrinkage to estimate fold changes, p-values and adjusted p-values obtained using the Benjamini-Hochberg method [29] for each filtered gene under the null hypothesis of common expression intensity across groups. Genes with adjusted p-values less than 0.10 were declared significant.

#### Differential exon usage analysis

We used the R package DEXSeq, version 1.6 (http://www.bioconductor.org/packages/release/bioc/html/DEXSeq.html), to perform differential exon usage analysis of *Sf3b1*^+/−^ versus *Sf3b1*^+/+^ samples. DEXSeq uses a negative binomial (NB) distribution to model the exon read counts and shrinkage estimators to estimate the per-exon NB dispersion parameters. We defined a testable exon as one that had a total sum of at least 8 mapped reads across samples and was in a gene with no more than 70 exons. Before exon usage testing, we dropped any exons that were not testable or were in genes with less than 2 testable exons to improve testing power while maintaining type I error rates. We used the testForDEU function, which compares deviances from generalized linear model fits (assuming NB likelihood) to a chi-squared reference distribution, to estimate p-values and adjusted p-values obtained using the Benjamini-Hochberg method for each exon under the null hypothesis of common usage across groups. Exons with adjusted p-values less than 0.10 were considered significant. Logarithm base 2 fold changes (*Sf3b1*^+/−^/ *Sf3b1*^+/+^) for each exon were estimated using the function estimatelog2FoldChanges.

#### Gene set differential expression analysis

We used CAMERA [30], Competitive Gene Set Test Accounting for Inter-Gene Correlation approach, as implemented in the camera function from the R package limma version 3.17.17, on TMM normalized and voom weighted expression data to test whether a set of genes was highly ranked relative to other genes in terms of differential expression, accounting for inter-gene correlation. We mapped mouse gene symbols to human gene symbols using the MGI mouse to human homology mappings (http://www.informatics.jax.org/homology.shtml). We used the MSigDB database [31] version 4.0 (http://www.broadinstitute.org/gsea/msigdb/index.jsp), gene set collections c1 through c7 in our gene set analyses. For each MSigDB collection of gene sets, we ran the camera function to estimate p-values for the competitive null hypothesis that the genes in the tested gene set didn’t show stronger average differential expression relative to all tested genes not in the gene set. Adjusted p-values were calculated on the gene-set p-values per collection using the Benjamini-Hochberg method to control for the number of gene sets tested within a collection. Any gene sets with adjusted p-values less than 0.10 were declared significant.

### Statistical analysis

Comparison of hematologic parameters between *Sf3b1*^+/−^ and *Sf3b1*^+/+^ mice were analyzed using two-sample Wilcoxon signed rank test and presented as mean ± standard deviations. Statistical analyses were performed using R (www.r-project.org). Data were considered statistically significant if the *P* value was ≤ 0.05.

## Additional files

Additional file 1: Figure S1. Genotyping of wild type (*Sf3b1* ^+/+^) and *Sf3b1* haploinsufficient (*Sf3b1* ^+/−^) mice. Genomic DNA was extracted from tails and toes of *Sf3b1* pups after rederivation and subjected to PCR amplification by using specific primers as described in Methods. Examples of *Sf3b1* ^+/−^ mice (#1, 2, 4, and 7) identified by the presence of amplicons corresponding to wild type (WT: 1.5 Kb) and knock-out (KO: 0.9 Kb) alleles on a 1.2% agarose gel. Lane marked with MM indicates 1Kb Plus DNA ladder. Additional file 2: Figure S2.
*Sf3b1* ^+/−^ have no difference in the mast cell compartment compared to *Sf3b1* ^+/+^ mice. Immunohistochemistry (IHC) was used to evaluate the presence of mast cells in *Sf3b1* ^+/−^ (n = 2) and *Sf3b1* ^+/+^ (n = 2). Bone marrow cells (5 x 10^5^) were spotted on cytospin slides and IHC for CD117 (c-Kit) was performed. Additional file 3: Figure S3.
*Sf3b1* ^+/−^ mice have no difference in bone marrow cellularity compared to *Sf3b1* ^+/+^ mice. (a) Hematoxylin/eosin (H&E) was performed on bone marrow (BM) cells (3–5 × 10^5^) from *Sf3b1* ^+/−^ (n = 3) and *Sf3b1* ^+/+^ (n = 3). An H&E representative image showed normal trilineage hematopoiesis and no changes in BM cellularity in both groups of mice. (b) A bar graft shows mean ± standard deviations of the number of BM cells at the end of the follow-up between *Sf3b1* ^+/−^ (n = 4) and *Sf3b1* ^+/+^ (n = 3) mice. Additional file 4: Figure S4.
*Sf3b1* ^+/−^ mice have dyserythropoietic features in the bone marrow. Bone marrow (BM) cells (3–5 × 10^5^) were spotted on cytospin slides and iron stain (Prussian blue) was performed according to common pathology stain’s protocols. A representative image taken by light microscopy shows that BM cells from *Sf3b1* ^+/−^ showed specific dyserythropoietic features such as nuclear budding or nuclear irregularity (black arrows) that were not seen in *Sf3b1* ^+/−^ mice. Additional file 5: Figure S5.
*Sf3b1* ^+/−^ mice have ring sideroblasts in the bone marrow. Iron stain (Prussian blue) was performed on bone marrow (BM) cells (3–5 x 10^5^) derived from *Sf3b1* ^+/−^ and *Sf3b1* ^+/+^ mice. Images taken by light microscopy show presence of ring sideroblasts (black arrows) in the BM of *Sf3b1* ^+/−^ (n = 3) and absence in *Sf3b1* ^+/+^ (n = 2) mice. Additional file 6: Figure S6.
*Sf3b1* ^+/−^ mice do not have any liver abnormalities. Representative images from Hematoxylin & Eosin stain of liver sections from 2 *Sf3b1* ^+/−^ and 2 *Sf3b1* ^+/+^ mice show absence of hepatomegaly or abnormalities in the liver. Additional file 7: Table S1. Differential gene level between *Sf3b1* ^+/−^ and *Sf3b1* ^+/+^ mice. The expression level of all genes is presented as mean fold change between *Sf3b1* ^+/−^ and *Sf3b1* ^+/+^ mice. In total 10330 genes were found in the comparison *Sf3b1* ^+/−^ versus *Sf3b1* ^+/+^ mice. Additional file 8: Table S2. Comparison of hematopoietic-related gene sets between *Sf3b1* ^+/−^ and *Sf3b1* ^+/+^ mice. Gene set analysis shows hematopoietic –related genes between both groups of mice. Additional file 9: Table S3. Gene clustered in hematopoietic-related gene sets between *Sf3b1* ^+/−^ and *Sf3b1* ^+/+^ mice. The genes related to the specific gene set are summarized. Additional file 10: Figure S7.
*Sf3b1* ^+/−^ mice have features of low risk Myelodysplastic syndrome with ring sideroblasts. A table illustrating the criteria established by the hematopathology subcommittee of the *Mouse Models of Human Cancers Consortium* establishing the myeloid dysplasia in mice. The table has been adopted by Beachy, SM et al. (*Hematol Oncol Clin North Am* 2010) to summarize all the criteria and it shows the specific features harbored by the *Sf3b1* ^+/−^ mice (red color).

## Abbreviations

SF3B1: Splicing factor 3b, subunit 1
MDS: Myelodysplastic syndromes
MDS/MPN: Myelodysplastic/ Myeloproliferative neoplasms
AML: Acute myeloid leukemia
MCV: Mean corpuscular volume
PLTs: Platelets
WBC: White blood cells
Hgb: Hemoglobin
RBC: Red blood cells
RS: Ring sideroblasts
BM: Bone marrow
EMH: Extramedullary hematopoiesis
JAK2: Janus kinase 2
TET2: Ten-eleven translocation-2
MPL: Myeloproliferative leukemia virus oncogene
ALAS2: Aminolevulinate, Delta-, Synthase 2
ABCB7: ATP-binding cassette, sub-family B (MDR/TAP), Member 7
U2AF65: U2 small nuclear RNA auxiliary factor 2
SF3b14: Splicing factor 3B, 14 kDa subunit
EZH2: Enhancer of zeste homolog 2
RARS: Refractory anemia with ring sideroblasts
RARS-T: Refractory anemia with ring sideroblasts and marker thrombocytosis
PCR: Polymerase chain reaction
H&E: Hematoxylin & Eosin

## References

[1] Swerdlow SH, Campo E, Harris NE, Jaffe ES, Pileri SA, Stein H, Thiele J, Vardiman JW, Hasserjian RP, Gattermann N, Bennett JM, Brunning RD, Thiele J (2008). WHO classification of tumours of haematopoietic and lymphoid tissues. Refractory Anaemia with Ring Sideroblasts.

[2] Szpurka H, Tiu R, Murugesan G, Aboudola S, Hsi ED, Theil KS, Sekeres MA, Maciejewski JP (2006). Refractory anemia with ringed sideroblasts associated with marked thrombocytosis (RARS-T), another myeloproliferative condition characterized by JAK2 V617F mutation. Blood.

[3] Ceesay MM, Lea NC, Ingram W, Westwood NB, Gaken J, Mohamedali A, Cervera J, Germing U, Gattermann N, Giagounidis A, Garcia-Casado Z, Sanz G, Mufti GJ (2006). The JAK2 V617F mutation is rare in RARS but common in RARS-T. Leukemia.

[4] Flach J, Dicker F, Schnittger S, Kohlmann A, Haferlach T, Haferlach C (2010). Mutations of JAK2 and TET2, but not CBL are detectable in a high portion of patients with refractory anemia with ring sideroblasts and thrombocytosis. Haematologica.

[5] Hellstrom-Lindberg E, Cazzola M (2008). The role of JAK2 mutations in RARS and other MDS. Hematol Am Soc Hematol Educ Program.

[6] Boultwood J, Pellagatti A, Nikpour M, Pushkaran B, Fidler C, Cattan H, Littlewood TJ, Malcovati L, Della Porta MG, Jädersten M, Killick S, Giagounidis A, Bowen D, Hellström-Lindberg E, Cazzola M, Wainscoat JS (2008). The role of the iron transporter ABCB7 in refractory anemia with ring sideroblasts. PLoS One.

[7] Pellagatti A, Cazzola M, Giagounidis AA, Malcovati L, Porta MG, Killick S, Campbell LJ, Wang L, Langford CF, Fidler C, Oscier D, Aul C, Wainscoat JS, Boultwood J (2006). Gene expression profiles of CD34+ cells in myelodysplastic syndromes: involvement of interferon-stimulated genes and correlation to FAB subtype and karyotype. Blood.

[8] Yoshida K, Sanada M, Shiraishi Y, Nowak D, Nagata Y, Yamamoto R, Sato Y, Sato-Otsubo A, Kon A, Nagasaki M, Chalkidis G, Suzuki Y, Shiosaka M, Kawahata R, Yamaguchi T, Otsu M, Obara N, Sakata-Yanagimoto M, Ishiyama K, Mori H, Nolte F, Hofmann WK, Miyawaki S, Sugano S, Haferlach C, Koeffler HP, Shih LY, Haferlach T, Chiba S, Nakauchi H (2011). Frequent pathway mutations of splicing machinery in myelodysplasia. Nature.

[9] Papaemmanuil E, Cazzola M, Boultwood J, Malcovati L, Vyas P, Bowen D, Pellagatti A, Wainscoat JS, Hellstrom-Lindberg E, Gambacorti-Passerini C, Godfrey AL, Rapado I, Cvejic A, Rance R, McGee C, Ellis P, Mudie LJ, Stephens PJ, McLaren S, Massie CE, Tarpey PS, Varela I, Nik-Zainal S, Davies HR, Shlien A, Jones D, Raine K, Hinton J, Butler AP, Teague JW (2011). Somatic SF3B1 mutation in myelodysplasia with ring sideroblasts. N Engl J Med.

[10] Visconte V, Makishima H, Jankowska A, Szpurka H, Traina F, Jerez A, O'Keefe C, Rogers HJ, Sekeres MA, Maciejewski JP, Tiu RV (2012). SF3B1, a splicing factor is frequently mutated in refractory anemia with ring sideroblasts. Leukemia.

[11] Visconte V, Tabarroki A, Rogers HJ, Hasrouni E, Traina F, Makishima H, Hamilton BK, Liu Y, O'Keefe C, Lichtin A, Horwitz L, Sekeres MA, Hsieh FH, Tiu RV (2013). SF3B1 mutations are infrequently found in non-myelodysplastic bone marrow failure syndromes and mast cell diseases but, if present, are associated with the ring sideroblast phenotype. Haematologica.

[12] Makishima H, Visconte V, Sakaguchi H, Jankowska AM, Abu Kar S, Jerez A, Przychodzen B, Bupathi M, Guinta K, Afable MG, Sekeres MA, Padgett RA, Tiu RV, Maciejewski JP (2012). Mutations in the spliceosome machinery, a novel and ubiquitous pathway in leukemogenesis. Blood.

[13] Maciejewski JP, Padgett RA (2012). Defects in spliceosomal machinery: a new pathway of leukaemogenesis. Br J Haematol.

[14] Visconte V, Makishima H, Maciejewski JP, Tiu RV (2012). Emerging roles of the spliceosomal machinery in myelodysplastic syndromes and other hematological disorders. Leukemia.

[15] Visconte V, Rogers HJ, Singh J, Barnard J, Bupathi M, Traina F, McMahon J, Makishima H, Szpurka H, Jankowska A, Jerez A, Sekeres MA, Saunthararajah Y, Advani AS, Copelan E, Koseki H, Isono K, Padgett RA, Osman S, Koide K, O'Keefe C, Maciejewski JP, Tiu RV (2012). SF3B1 haploinsufficiency leads to formation of ring sideroblasts in myelodysplastic syndromes. Blood.

[16] Isono K, Mizutani-Koseki Y, Komori T, Schmidt-Zachmann MS, Koseki H (2005). Mammalian polycomb-mediated repression of Hox genes requires the essential spliceosomal protein Sf3b1. Genes Dev.

[17] Beachy SH, Aplan PD (2010). Mouse models of myelodysplastic syndromes. Hematol Oncol Clin North Am.

[18] Wang L, Lawrence MS, Wan Y, Stojanov P, Sougnez C, Stevenson K, Werner L, Sivachenko A, DeLuca DS, Zhang L, Zhang W, Vartanov AR, Fernandes SM, Goldstein NR, Folco EG, Cibulskis K, Tesar B, Sievers QL, Shefler E, Gabriel S, Hacohen N, Reed R, Meyerson M, Golub TR, Lander ES, Neuberg D, Brown JR, Getz G, Wu CJ (2011). SF3B1 and other novel cancer genes in chronic lymphocytic leukemia. N Engl J Med.

[19] Mian SA, Rouault-Pierre K, Smith AS, Seidl T, Kulasekararaj AG, Mohamedali AM, Shinde S, Bonnet D, Mufti GJ (2013). SF3B1 mutant clones from patients with refractory anaemia with ringed sideroblasts (RARS) originate from the early haematopoietic stem cells and maintain their engraftment potential. Blood (ASH Annual Meeting).

[20] Ramirez-Herrick AM, Mullican SE, Sheehan AM, Conneely OM (2011). Reduced NR4A gene dosage leads to mixed myelodysplastic/myeloproliferative neoplasms in mice. Blood.

[21] Wegrzyn J, Lam JC, Karsan A (2011). Mouse models of myelodysplastic syndromes. Leuk Res.

[22] Matsunawa M, Yamamoto R, Sanada M, Sato-Otsubo A, Shiozawa Y, Yoshida K, Otsu M, Shiraishi Y, Miyano S, Isono K, Koseki H, Nakauchi H, Ogawa S (2014). Haploinsufficiency of Sf3b1 leads to compromised stem cell function but not to myelodysplasia. Leukemia.

[23] Sekeres MA, Schoonen WM, Kantarjian H, List A, Fryzek J, Paquette R, Maciejewski JP (2008). Characteristics of US patients with myelodysplastic syndromes: results of six cross-sectional physician surveys. J Natl Cancer Inst.

[24] Chen TC, Hou HA, Chou WC, Tang JL, Kuo YY, Chen CY, Tseng MH, Huang CF, Lai YJ, Chiang YC, Lee FY, Liu MC, Liu CW, Liu CY, Yao M, Huang SY, Ko BS, Hsu SC, Wu SJ, Tsay W, Chen YC, Tien HF (2014). Dynamics of ASXL1 mutation and other associated genetic alterations during disease progression in patients with primary myelodysplastic syndrome. Blood Cancer Journal.

[25] Wang J, Li Z, He Y, Pan F, Chen S, Rhodes S, Nguyen L, Yuan J, Jiang L, Yang X, Weeks O, Liu Z, Zhou J, Ni H, Cai CL, Xu M, Yang FC (2014). Loss of Asxl1 leads to myelodysplastic syndrome-like disease in mice. Blood.

[26] Watanabe-Okochi N, Kitaura J, Ono R, Harada H, Harada Y, Komeno Y, Nakajima H, Nosaka T, Inaba T, Kitamura T (2008). AML1 mutations induced MDS and MDS/AML in a mouse BMT model. Blood.

[27] Robinson MD, Oshlack A (2010). A scaling normalization method for differential expression analysis of RNA-seq data. Genome Biol.

[28] Law CW, Chen Y, Shi W, Smyth GK (2014). Voom: precision weights unlock linear model analysis tools for RNA-seq read counts. Genome Biol.

[29] Benjamini YHY (1995). Controlling the false discovery rate: a practical and powerful approach to multiple testing. J R Statis Soc B.

[30] Wu D, Smyth GK (2012). Camera: a competitive gene set test accounting for inter-gene correlation. Nucleic Acids Res.

[31] Liberzon A, Subramanian A, Pinchback R, Thorvaldsdottir H, Tamayo P, Mesirov JP (2011). Molecular signatures database (MSigDB) 3.0. Bioinformatics.

