# Supplementary figures and images for: Splicing factor 3b subunit 1 (Sf3b1) haploinsufficient mice display features of low risk Myelodysplastic syndromes with ring sideroblasts
Source: J Hematol Oncol. 2014 Dec 7;7:89. doi: 10.1186/s13045-014-0089-x (PMC4266210; doi:10.1186/s13045-014-0089-x)

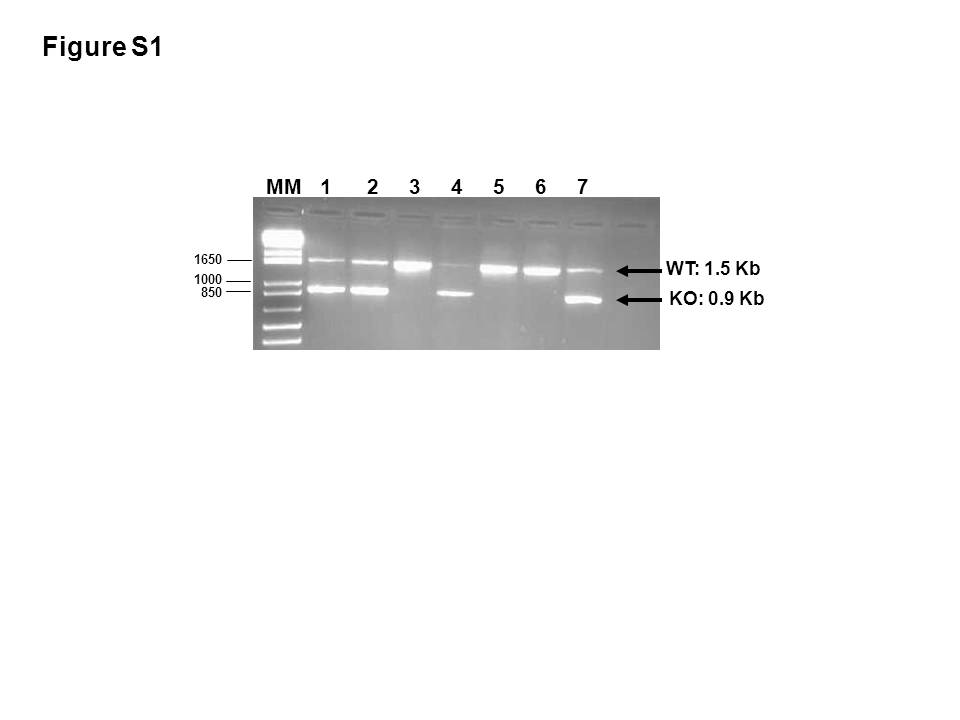

Supplement: Additional file 1: Figure S1. — Genotyping of wild type (Sf3b1 +/+) and Sf3b1 haploinsufficient (Sf3b1 +/−) mice. Genomic DNA was extracted from tails and toes of Sf3b1 pups after rederivation and subjected to PCR amplification by using specific primers as described in Methods. Examples of Sf3b1 +/− mice (#1, 2, 4, and 7) identified by the presence of amplicons corresponding to wild type (WT: 1.5 Kb) and knock-out (KO: 0.9 Kb) alleles on a 1.2% agarose gel. Lane marked with MM indicates 1Kb Plus DNA ladder. [file 13045_2014_89_MOESM1_ESM.jpeg]

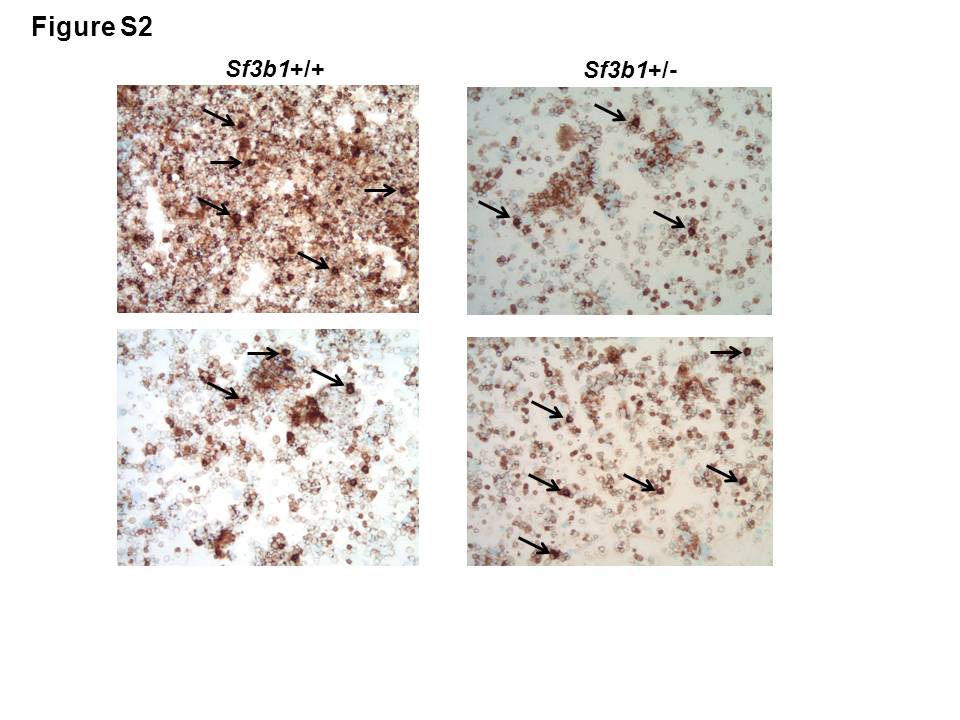

Supplement: Additional file 2: Figure S2. — Sf3b1 +/− have no difference in the mast cell compartment compared to Sf3b1 +/+ mice. Immunohistochemistry (IHC) was used to evaluate the presence of mast cells in Sf3b1 +/− (n = 2) and Sf3b1 +/+ (n = 2). Bone marrow cells (5 x 10^5^) were spotted on cytospin slides and IHC for CD117 (c-Kit) was performed. [file 13045_2014_89_MOESM2_ESM.jpeg]

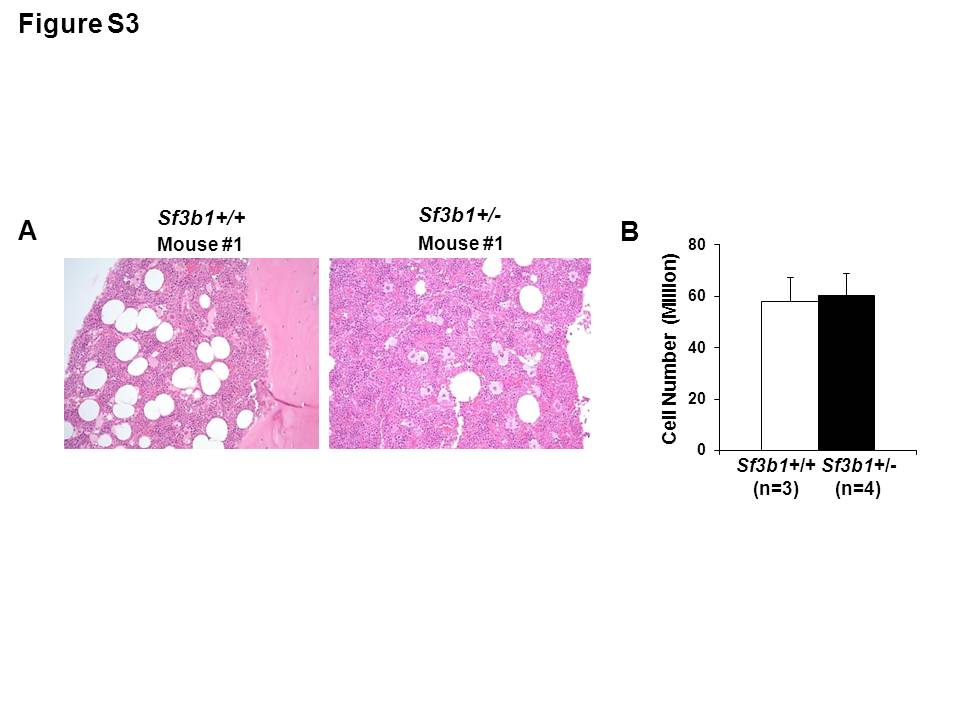

Supplement: Additional file 3: Figure S3. — Sf3b1 +/− mice have no difference in bone marrow cellularity compared to Sf3b1 +/+ mice. (a) Hematoxylin/eosin (H&E) was performed on bone marrow (BM) cells (3–5 × 10^5^) from Sf3b1 +/− (n = 3) and Sf3b1 +/+ (n = 3). An H&E representative image showed normal trilineage hematopoiesis and no changes in BM cellularity in both groups of mice. (b) A bar graft shows mean ± standard deviations of the number of BM cells at the end of the follow-up between Sf3b1 +/− (n = 4) and Sf3b1 +/+ (n = 3) mice. [file 13045_2014_89_MOESM3_ESM.jpeg]

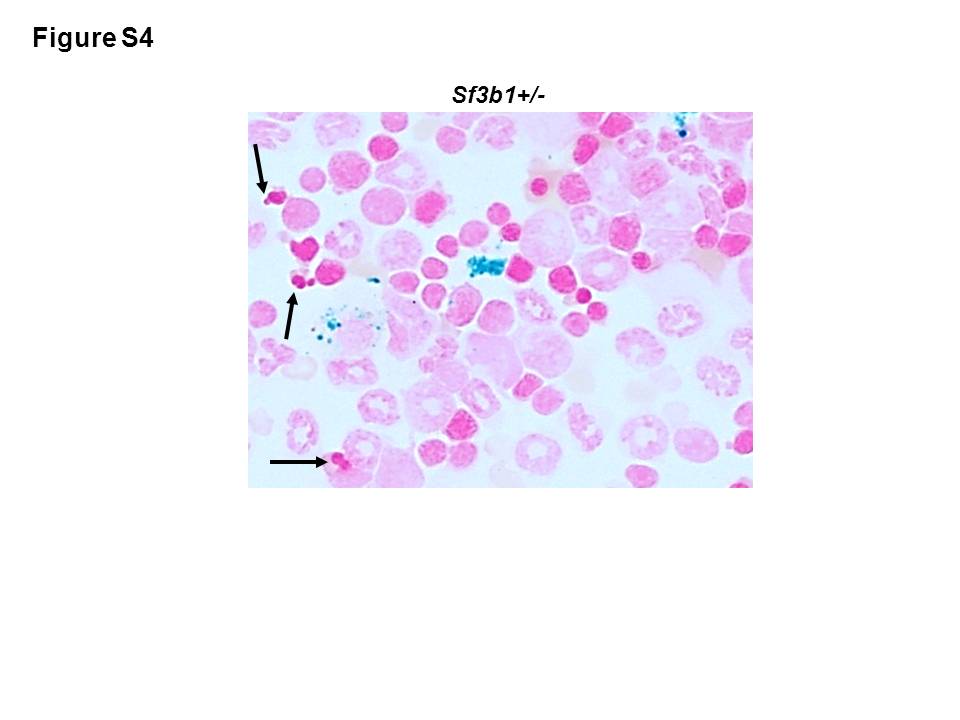

Supplement: Additional file 4: Figure S4. — Sf3b1 +/− mice have dyserythropoietic features in the bone marrow. Bone marrow (BM) cells (3–5 × 10^5^) were spotted on cytospin slides and iron stain (Prussian blue) was performed according to common pathology stain’s protocols. A representative image taken by light microscopy shows that BM cells from Sf3b1 +/− showed specific dyserythropoietic features such as nuclear budding or nuclear irregularity (black arrows) that were not seen in Sf3b1 +/− mice. [file 13045_2014_89_MOESM4_ESM.jpeg]

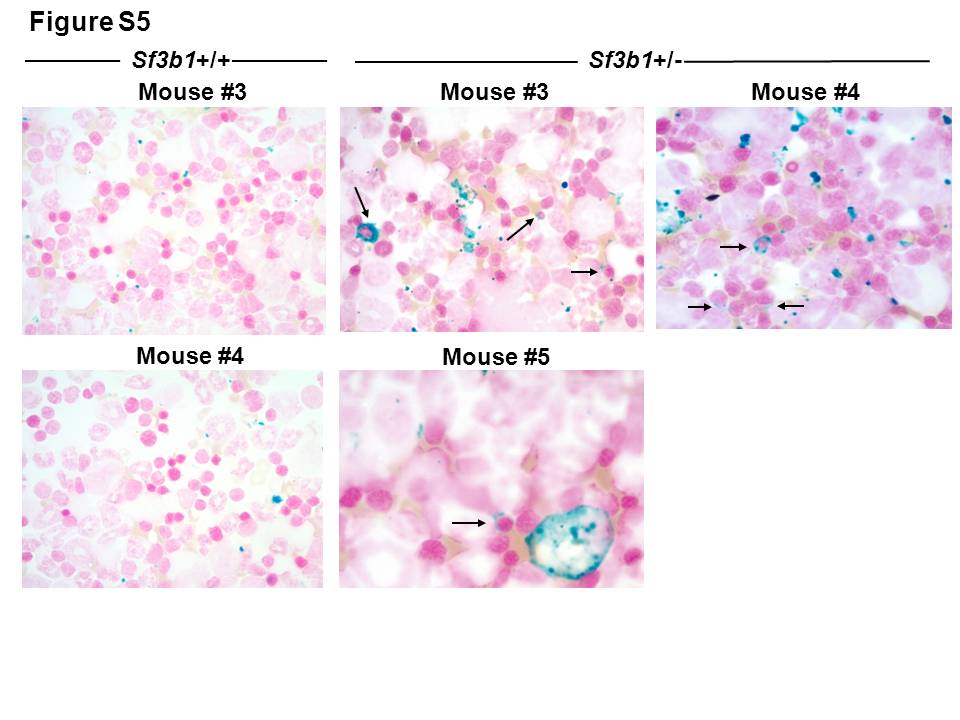

Supplement: Additional file 5: Figure S5. — Sf3b1 +/− mice have ring sideroblasts in the bone marrow. Iron stain (Prussian blue) was performed on bone marrow (BM) cells (3–5 x 10^5^) derived from Sf3b1 +/− and Sf3b1 +/+ mice. Images taken by light microscopy show presence of ring sideroblasts (black arrows) in the BM of Sf3b1 +/− (n = 3) and absence in Sf3b1 +/+ (n = 2) mice. [file 13045_2014_89_MOESM5_ESM.jpeg]

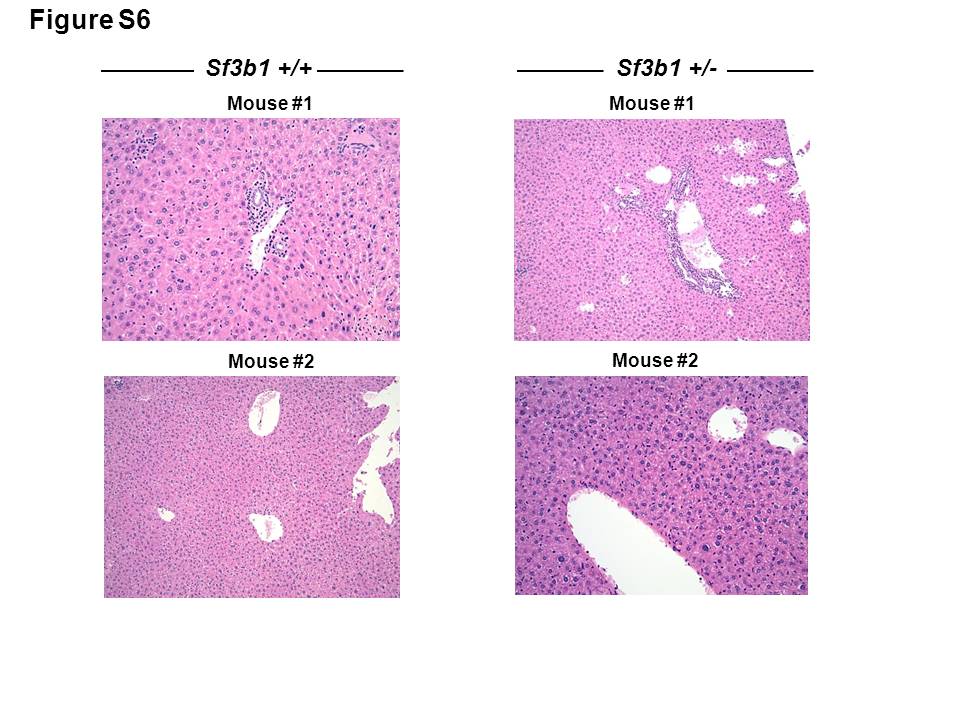

Supplement: Additional file 6: Figure S6. — Sf3b1 +/− mice do not have any liver abnormalities. Representative images from Hematoxylin & Eosin stain of liver sections from 2 Sf3b1 +/− and 2 Sf3b1 +/+ mice show absence of hepatomegaly or abnormalities in the liver. [file 13045_2014_89_MOESM6_ESM.jpeg]

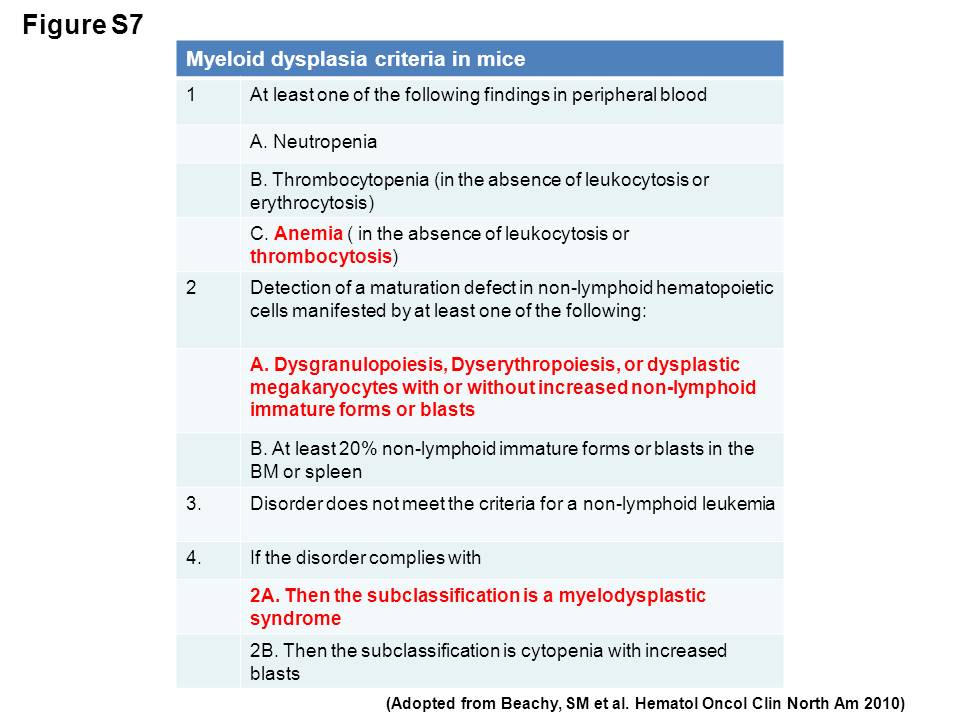

Supplement: Additional file 10: Figure S7. — Sf3b1 +/− mice have features of low risk Myelodysplastic syndrome with ring sideroblasts. A table illustrating the criteria established by the hematopathology subcommittee of the Mouse Models of Human Cancers Consortium establishing the myeloid dysplasia in mice. The table has been adopted by Beachy, SM et al. (Hematol Oncol Clin North Am 2010) to summarize all the criteria and it shows the specific features harbored by the Sf3b1 +/− mice (red color). [file 13045_2014_89_MOESM10_ESM.jpeg]
